# Effects of atrial tachycardia and dehydration in an elderly patient with a leadless ventricular pacemaker implantation: A case report

**DOI:** 10.1016/j.hrcr.2022.11.006

**Published:** 2022-11-17

**Authors:** Naoya Inoue, Takashi Ogane, Shuji Morikawa

**Affiliations:** ∗ Department of Cardiology, Chutoen General Medical Center, Kakegawa, Japan; † Department of Cardiology, Nagoya University Graduate School of Medicine, Nagoya, Japan

**Keywords:** Atrial tachycardia, Atrioventricular synchrony, Dehydration, Left ventricular inflow flow velocity waveform, Micra AV

## Introduction

Recently, leadless pacemakers such as the Micra (Medtronic) have become popular among patients who require pacemakers. Among Micra pacemakers, the Micra AV (Medtronic, Minato Ward, Tokyo, Japan) with VDD mode is used to maintain atrioventricular synchrony (AVS) in patients with an atrioventricular block (AVB). Complications associated with conventional transvenous pacemakers (TVPs)—such as device infection and subclavian vein stenosis—remain a concern, particularly in thin, elderly patients. The safety of Micra implantation in the elderly has already been reported.^1^ Additionally, although the Micra AV can provide AVS and can reduce pacemaker complications and maintain cardiac output,^2^ atrial mechanical sensing (AMS) using this device has various limitations.

**Key Teaching Points**

• When enhancing atrioventricular synchrony with the Micra AV (Medtronic), the possibility of dehydration and supraventricular tachycardia should be considered.
• The A3/A4 signal is closely related to ventricular inflow velocity, and a threshold should be set when evaluating the E/A ratio.
• To ensure adequate cardiac output, indications for the Micra AV should be carefully considered in frail and elderly patients, as they are more prone to dehydration.

The feature of this pacemaker is that it does not have an atrial lead; thus, mechanical sensing of the blood flow owing to atrial contraction (known as the A4 signal) provides appropriately timed ventricular pacing (VP). Pacemakers that do not maintain AVS are known to cause reduced cardiac output; therefore, considering that this is the right VP, it is suggested that implantation of the Micra AV may be disadvantageous. In this case report, a patient with AVB underwent Micra AV implantation and later developed atrial tachycardia (AT). Therefore, we set the A4 threshold to allow AMS; however, after discharge from the hospital, the patient developed activities of daily living decline and worsening dehydration, and the Micra AV synchrony could not be maintained. This report highlights the need to consider whether the Micra AV is appropriate for use in elderly patients with AVB who have decreased activity.

## Case report

**Figure 1:**
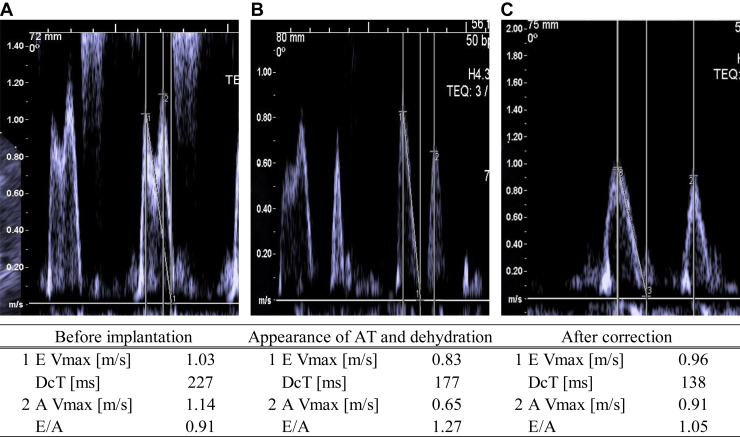
Evaluation of E/A ratio by echocardiography. **A:** Before Micra AV (Medtronic) implantation. **B:** Appearance of atrial tachycardia and after dehydration. **C:** After correction for dehydration by intravenous infusion. AV = atrioventricular; E/A = Emax/Amax.

A 96-year-old woman presented with chronic heart failure due to aortic stenosis, hypertension, and chronic kidney disease due to nephrosclerosis. Approximately 6 months after first visiting the hospital, the patient was rushed to the emergency room owing to loss of consciousness. An electrocardiogram (ECG) at the time of visit showed complete AVB, and blood tests on the same day revealed a uric acid (UA) level of 6.9 mg/dL, urea nitrogen (UN) level of 45.5 mg/dL, creatinine level of 2.1 mg/dL, and brain natriuretic protein (BNP) level of 319 pmol/L. Echocardiography (UCG) revealed preserved left ventricular ejection fraction (55%); Emax, 1.03 m/s; Amax, 1.14 m/s (E/A: 0.91); deceleration time, 227 ms; E': 4.2 cm/s (Figure 1A). Physical examination revealed fluid retention, including pitting edema and jugular vein distention, and the patient was admitted to the hospital with heart failure due to AVB.

The patient’s advanced age and the need for pacemaker implantation as a therapeutic intervention for the underlying disease were thoroughly discussed with the patient’s family. The patient strongly desired this treatment; however, owing to her small build (height: 150.0 cm, weight: 40.8 kg, body mass index: 18.1 kg/m^2^) and the risk of infection owing to advanced age, the Micra AV was selected instead of a TVP. The Micra AV was implanted in the right ventricular septum without any complications, and the pacemaker data were within normal parameters (threshold: 0.25 V / 0.24 ms, sensing: 14.5 mV, and resistance: 960 Ω). During the manual atrial mechanical (MAM) test after implantation, AT was observed (atrial rate: about 180 beats/min). When the A3/A4 threshold was set to 4.5 m/s^2^ and 1.2 m/s^2^, respectively, good AMS and VP were obtained, and AVS was maintained.

**Figure 2:**
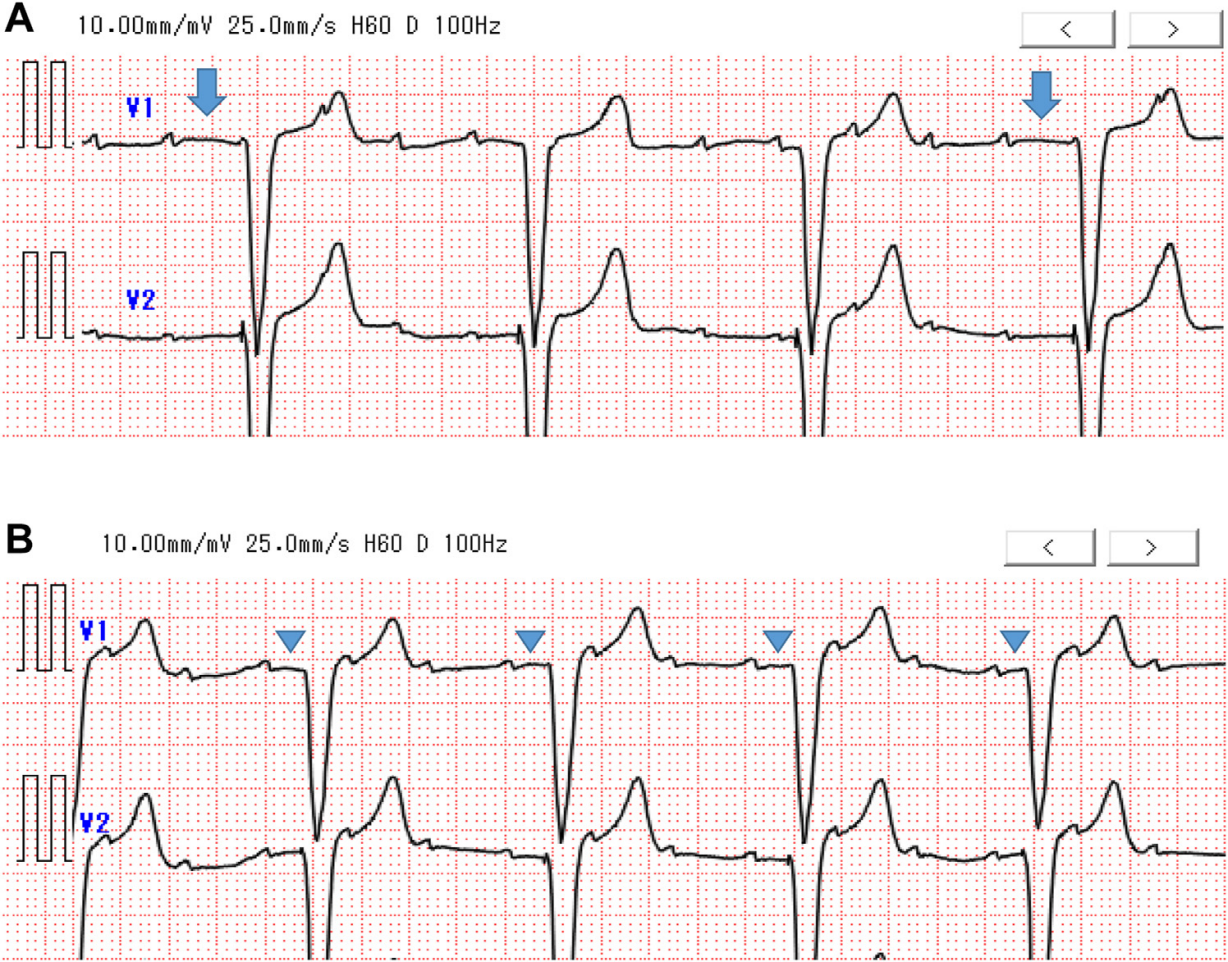
Evaluation of atrioventricular synchrony by electrocardiography. **A:** After atrial tachycardia and dehydration. **B:** After correction for dehydration by intravenous infusion. Arrow indicates a paced ventricular beat more than 300 ms from the electrocardiography-confirmed P wave. Arrowhead indicates a regularly paced ventricular beat within 300 ms of the P wave.

**Figure 3:**
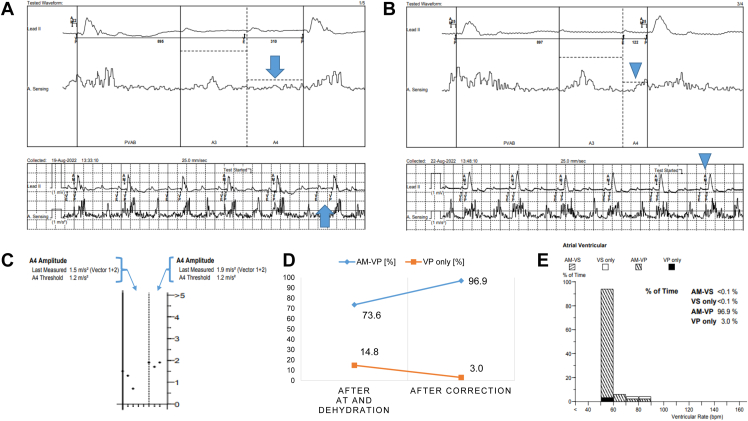
Manual atrial mechanical test. **A:** After atrial tachycardia and dehydration. **B:** After correction for dehydration. **C:** Comparison of A4 amplitude after dehydration (left panel) and correction (right panel). **D:** The transition of atrial mechanical sensing-ventricular pacing (AM-VP) and ventricular pacing (VP)-only rates. **E:** Histogram of AM-VP and VP only after dehydration correction. Arrow indicates undersensing and no atrioventricular synchrony (AVS). Arrowhead indicates A4 signal is good and AVS is obtained.

One week after discharge, the patient was rushed back to our hospital owing to weakness caused by dehydration. Blood tests (UA level: 11.9 mg/dL, UN: 71.6 mg/dL, creatinine: 1.9 mg/dL, BNP: 126 pmol/L) and second UCG were performed, and no significant change in left ventricular contractility was observed (left ventricular ejection fraction: 61%). Changes were observed in the left ventricular inflow velocity waveforms: Emax, 0.83 m/s; Amax, 0.65 m/s (E/A: 1.27); deceleration time, 177 ms; and E', 3.7 cm/s (Figure 1B). ECG and MAM tests were performed; the ECG revealed persistent AT, and the interval between AMS and VP was irregular (Figure 2A). The MAM test indicated an undersensing of the A4 signal (Figure 3A); therefore, we resolved to treat the patient by administering supplemental fluids. Four days after supplementation, blood tests showed that dehydration had improved (UA level: 8.5 mg/dL, UN: 46.5 mg/dL, creatinine: 1.9 mg/dL, and BNP: 123 pmol/L); ECG, UCG, and MAM test results were reevaluated. The ECG results demonstrated that AVS was maintained (Figure 2B), and UCG showed improvement in the left ventricular inflow velocity wave and a decrease in the E/A ratio (Emax: 0.96 m/s, Amax: 0.91 m/s, and E/A 1.05; Figure 1C). The MAM test also showed that the A4 signal was sensed above the threshold (Figure 3B and 3C), and good AMS and VP were confirmed again (Figure 3B–3E).

## Discussion

The Micra AV is a revolutionary pacemaker that enables mechanical sensing of atrial waves and appropriately timed VP by simply implanting the pacemaker in the ventricle. However, concerns about cardiac accidents associated with the Micra have been reported since its introduction, and maintaining perfect AVS with the Micra AV remains a challenge; the synchronization rate was reported to be approximately 70%–80%, depending on the patient’s resting, exertion, and posture.^3^

The importance of AV synchronization in pacemakers has been previously reported.^4^ A previous report demonstrated a statistically significant improvement in velocity-time integral compared with VVI and VDD using the Micra AV.^2^ Increasing stroke volume or cardiac output is particularly important in patients with heart failure; therefore, DDD-TVP is recommended when possible to achieve AVS. Nonetheless, the Micra AV is an important option for patients with background conditions, such as pacemaker infection or blood access problems owing to hemodialysis.

One prediction of AV synchrony in VDD correlates with the E/A ratio as reported by Garweg and colleagues.^5^ In VDD, the A3 signal correlates with the E wave (the early diastolic wave of left ventricular inflow) and the A4 signal with the A wave (the atrial systolic wave).^5^ Previous reports have shown that a high AVS rate can be obtained when the E/A ratio is <0.94^5^; in this case, the preimplantation echocardiographic results predicted a high degree of AVS with VDD. In fact, the MAM test, which was performed immediately after implantation, demonstrated good AVS; however, subsequent dehydration may have led to the breakdown of interventricular synchrony. It is assumed that frequent atrial contraction due to AT reduced atrial contractility and the accompanying blood flow rate from the atria to the ventricles, which in turn reduced the single cardiac output. Additionally, the reduced venous perfusion owing to dehydration may have further reduced blood flow into the right ventricle, resulting in mechanical sensing failure. As evidence for this mechanism, a comparison of the E wave, A wave, and E/A ratio before dehydration, after dehydration, and after correction for dehydration showed that the left ventricular inflow velocity wave and E/A ratio changed. Consistent with this, while the A4 threshold was lower for AT in the MAM test, the effect of dehydration still caused the A4 signal to be poorly sensed and AVS to fail.

For such conditions, catheter ablation therapy for AT is one of the promising methods to improve AVS, but in this patient’s case we decided to forego further invasive treatment because of her advanced age. It is also suggested that adequate hydration improves atrial pressure waveforms and AMS. Therefore, in this case, the AV ratio was set to follow the tachycardia at an AV ratio of 3:1 to maintain AVS as much as possible. However, the limitation is that the AT rate must not fluctuate, and if the cycle length fluctuates depending on whether focal AT or reentrant AT is used, there may be a risk of being unable to maintain AVS. Particularly, it was difficult to accurately evaluate the effect of dehydration on the atrial rate and cycle length; therefore, maintaining AVS in such conditions is a future challenge.

As mentioned above, the Micra is a revolutionary device that improves the complications of conventional pacemakers. Still, the Micra AV should be carefully considered before its application, as its function may be affected by postimplantation events. Particularly, the Micra is currently recommended for less active rather than more active patients because of its current performance. Of course, battery life, device replacement, and additional devices, in the case of Micra, are required; however, from this perspective, the Micra may be suitable for the elderly at this time. However, it should be emphasized that the elderly, who are prone to activities of daily living decline and consequent dehydration, may not benefit from Micra AV because dehydration may reduce AMS owing to reduced blood flow into the ventricles. Conversely, the difficulty in tracking an AT may be encountered by both conventional TVP and Micra AV.

## Conclusion

This study reports the case of an elderly patient with AVB who was predicted to have good AVS based on UCG; however, dehydration and AT events led to a loss of AVS. We strongly recommend that the decision to use the Micra AV be based on possible changes in the patient’s condition after implantation, not on the current activity assessment.
